# Evaluation of different agroindustrial waste on the effect of different carcass characteristics and physiological and biochemical parameters in broilers chicken

**DOI:** 10.14202/vetworld.2017.368-374

**Published:** 2017-04-03

**Authors:** Y. Sánchez-Roque, Y. D. C. Pérez-Luna, E. Pérez-Luna, R. Berrones Hernández, S. Saldaña-Trinidad

**Affiliations:** 1 Department of Agroindustrial Engineering, Universidad Politécnica de Chiapas, Eduardo J. Selvas s. n. Col. Magisterial. C. P. 29080, Tuxtla Gutiérrez, Chiapas, México; 2 Department of Zootechnical, Faculty of Agronomic Sciences Campus V Universidad Autónoma de Chiapas, Carretera Ocozocoautla Villaflores, CHIS 230, 30470 Chis, Mexico

**Keywords:** agroindustrial waste, broiler chickens, feed conversion ratio, parameters

## Abstract

**Aim:**

This study was conducted to evaluate the physiological and biochemical effect in chickens of the Ross breed of a food enriched with agroindustrial waste.

**Materials and Methods:**

The food is one of the main components of the total cost for the production of chickens. Rations should be formulated to provide the correct balance of energy, protein, amino acids, minerals, vitamins and essential fatty acids, to allow optimal growth and performance. This study was intended to evaluate a natural feed for chicken, made from corn, yucca meal, eggshells, orange peel, soybean meal, salt and garlic, enriched with agroindustrial waste (molasses, milk whey and ferment of coffee). The weight gain was evaluated in broilers using a diet enriched with different agroindustrial wastes, with respect to a control food of the same composition but not containing residue. To develop the experiment 120 male Ross 308 chicks were used, these were evaluated for 6 weeks. Physicochemical test for the food and the agroindustrial waste were performed; moisture was determined; organic carbon, organic material and the ash, to characterize the agroindustrial wastes, the reducing sugars content using a spectrophotometer at 540 nm and proteins through the Kjeldahl method was evaluated. During the experiment, the weight gain of chickens and feed conversion was evaluated; the end of the experiment the weight of eviscerated channel relative weight breast, thighs, pancreas, and abdominal fat was determined, besides including blood chemistries as determination of cholesterol, triglycerides, and glucose. Finally, the microbiological analyzes to detect the presence of *Escherichia coli* in the cecum was determinate. Data were analyzed by InfoStat statistical program using the generalized linear model procedure. The statistical comparison was made by Tukey test at the 95% probability level.

**Results:**

After the evaluation, fed chickens with the treatments food + milk whey (FMW) and food + ferment of coffee (FFC) demonstrated increased from 1949 to 1892 g, respectively, to the 42 days of evaluation, showing the best treatment for weight gain. However, the FFC treatment showed the best feed conversion reaching values of 1.79 related to levels of blood glucose (249 mg/dl). Even so the eviscerated channel yields were higher for the treatment FFC reaching 1810.1 g unlike the treatment FMW which it reached a weight of 1718.2 g with increased formation of abdominal fat (7.4 g) unlike other treatments. From the results, it is concluded that food enriched with coffee ferment allows an increase in weight, better feed conversion in addition to high production of lean meat.

**Conclusion:**

It was shown that the best treatment was the food enriched with the ferment of coffee, due to increased intake and weight gain at the end of 42 days of the experiment.

## Introduction

The marketing of the broiler chicken production tops the meat industry, in its first efforts efficiently convert ingredients of animal and plant products in high quality food. The rearing of chickens, plus egg production are activities that are performed to produce as much at the lowest cost and to achieve this you need to combine three elements: Excellent genetic material, food that meets the nutritional requirements of the species, preventive management against diseases. If these three factors are combined properly the ultimate goal are: A healthy chicken, with good weight and good feed conversion [1,2].

Food is important to achieve maximum productive expression. The success achieved so far with this practice is for the better understanding of the roles of different nutrients, allowing with more accurately meet the nutritional needs. In the chicken feed, it is necessary to know the stages or phases of food to meet the nutritional requirements. These stages or phases are important for maximum utilization of food and nutrients. These divisions are based on physiological and metabolic processes of the animal and its goal is to provide to the chicken the necessary amount of nutrients required in a certain age, to avoid waste or overfeeding [3,4].

In recent years, it has been transformed the balanced diet in the poultry industry, because it has tried to change current commercial foods, as these represent a health issue because they use chemicals to supplement the nutrition of broilers such as hormones to accelerate their growth and profit regardless of the effects they may cause. At present, they have sought alternative food and debris that could contribute and complement the nutritional quality of foods intended for poultry [5-7].

Regarding costs, food is the most important for the production of poultry so that the availability of low-cost food and quality is essential for the expansion of the poultry industry. It is expected that in developing countries, increasing costs and decreasing supply of traditional foods limit the future expansion of poultry production. This situation highlights the urgent need to improve the use of the wide range of alternative foods available to these countries. In many cases, food resources are not used, wasted or used inefficiently. The use of most alternative foods is currently negligible due to limitations imposed by technical and socioeconomic factors [8].

Based on the aforementioned reasons, the waste of the agroindustrial processes are an important element that can alter the environmental balance and jeopardize the quality of life of human beings if they are not treated or exploited efficiently [9]. For this purpose currently are produced three agroindustrial waste that generated environmental problems and detonates on the health of people these are molasses from the sugar industry, fermentation of coffee generated in the wet processing of coffee and the whey generated by the dairy industry, the waste resulting from a process of agroindustrial processing has high concentrations of biomolecules needed to enrich smoothly balanced food such as water, carbohydrates, fats, proteins, vitamins and minerals, so it is thought that by enriching a food with these residues are sufficient for the chickens grow healthy and productive.

Therefore, the alternative poultry farming is inserted into the generation process technologies and production systems adapted to local ecosystems and to the specific characteristics of small producers, which are compatible with the sustainable management of natural resources. Hence, the aim of this study was to evaluate the physiological and biochemical effect in chickens Ross breed to enrich a food with agroindustrial waste.

## Materials and Methods

### Ethical approval

The procedures have been approved by Ethic Committees of the Authors’ Institutions’, and care was taken to minimize the number of animals used.

### Animals

Before the experiment, the poultry rearing unit was thoroughly cleaned for any leftover refuse from the previous batch. The building complex was then fumigated, washed, and disinfected with 0.8% formalin solution. Birds were reared in land cages of 1.5 m (length), 1.5 m (width), and 1.5 m (height). The rearing land cages, feeding and drinking units were installed and fumigated 72 h before introducing the broilers into the unit. 120 male Ross 308 chicks were purchased and transferred into the rearing area in a postnatal age, with a weight of 348-511 g. During the experimental period, the temperature of the building was thermostatically maintained using two gasoline rocket heaters. The air relative humidity was maintained at 60-65%. The aeration was provided by two fans. One feeding tray of plastic and one drinking water container per cage was used. For sanitation, drinkers were washed daily.

### Experimental design

The experimental feeding lasted for a total of 42 days, after which five birds per treatments, was slaughtered. The different treatments were set as following form (Table-1). The treatments are designed for a staple food consisting of corn, yucca meal, eggshells, orange peel, soybean meal, salt and garlic, enriched with agroindustrial wastes (molasses, milk whey and ferment of coffee), each treatment was evaluated with a total of 10 chickens by triplicate (evaluating a total of 30 chickens per treatment) during 1^st^ to 42^th^ day; as shown below:

**Table-1:** Feed control ingredients and nutrient analysis of used diets during the starter and finisher periods by 42^nd^ day.

Ingredient (%)	Starter and finisher period (postnatal 1^st^-42^nd^ day)				
Feed ingredients					
Soybean meal	35				
Corn	35				
Yucca meal	10				
Egg shell	8				
Orange peel	8				
Garlic	1				
Salt	2.7				
*Vitamin mixture	0.3				
**Physical and chemical analysis**					

	**FC**	**FM**	**FFC**	**FMW**	**SEM**

Humedad (%)	20.6^a^	21.5^a^	22.1^a^	22.7^a^	0.7**
Carbono orgánico (%)	54.2^a^	67.4^a^	62.1^a^	60.5^a^	4.7
Nutrient analysis					
Proteína (%)	26.4^a^	29.5^a^	40.6^b^	63.3^c^	18.1
Lysine (%)	1.47^a^	1.90^a^	2.94^b^	4.41^c^	1.13
Methionine (%)	0.57^a^	1^b^	1.14^b^	1.72^c^	0.41
Calcium (%)	1.15^a^	3.40^b^	1.60^a^	2.33^c^	0.85
Available phosphorus (%)	0.50^a^	0.65^a^	0.88^b^	1.01^b^	0.19

* Vitamin mixture: Vitamin A - 5000 IU/g, Vitamin D3 - 500 IU/g, Vitamin E - 3 mg/g, Vitamin K3 1.5 mg/g, Vitamin B2 1 mg/g.

** Means (±standard error) within each column of dietary treatments with no common superscript differ significantly at p<0.05. FC=Food control, FM=Food+molasses, FFC=Food+ferment of coffee, FMW=Food+milk whey

- Treatment 1: Basal diet - Food control, during 1^st^ to 42^th^ day
- Treatment 2: Basal diet - Food + molasses (FM), during 1^st^ to 42^th^ day
- Treatment 3: Basal diet - Food + ferment of coffee (FFC), during 1^st^ to 42^th^ day
- Treatment 4: Basal diet - Food + milk whey (FMW), during 1^st^ to 42^th^ day.

### Diets

The ingredient composition of the diets used as well as the nutrient composition is presented in Table-1. According to the chemical composition of the FM and FFC treatments are considered as isocaloric foods, and the FMW food is considered isoprotic. Feed and water were supplied daily by the experimental period. The nutritional requirements of the broilers were provided based on the Ross 308 strain rearing catalog.

### Performance

Feed intake and weight gain were recorded daily. Feed conversion ratio was calculated by Patel *et al*. [6].

### Physicochemical characterization of treatments

In preparation of samples for chemical analysis proximal, the moisture of the treatments was assessed by drying at 105°C for 24 h and organic carbon was determined by a muffle furnace at 550°C [10,11]. Crude protein was analyzed by Kjeldahl method [12,13]. Nutritional analysis was performed according to Alhotan *et al*. [14], who evaluate the percentage concentration of lysine, methionine, calcium and available phosphorus (Table-1).

### Physicochemical characterization of the agroindustrial waste

For characterization of agroindustrial wastes reducing sugars concentration, it was determined from the reduction of the acid 3-5 dinitrosalicylic (yellow) by reducing sugars, the acid 3-amino-5-salicylic nitro (brick red) detected at 540 nm using a spectrophotometer (ZUZI 4255/50), according to Chranioti *et al*. [15]. Thus, the potential of hydrogen (pH) with the use of a potentiometer (HANNA HI 2211-01) was also determined. According to Konca *et al*. [16], finally the total soluble solids using a refractometer (RHC-200ATC) was determined according to the established by Chavan *et al*. [17] and Skoog *et al*. [18] (Table-2).

**Table-2:** Physico-chemical characterization of agroindustrial waste.

Agroindustrial waste	pH	Acidity (mL of lactic acid equivalent)	Protein (%)	Reducing sugars (mg/mL)
Molasses	6.3^ª^	2.7^a^	3.1^a^	0.5^ª^
Ferment of coffee	3.6^b^	1.4^ª^	14.2^ª^	3.7^b^
Milk whey	5.3^c^	2.3^ª^	36.8^b^	2.4^b^
SEM*	1.1	0.5	1.5	1.3

* Means (±standard error) within each column of dietary treatments with no common superscript differ significantly at p<0.05. SEM: Standard error of mean

### Biochemical parameters

At the end of the study, on the postnatal 42^th^ day, three birds per group, totaling 4 birds per treatment, was selected for blood sampling. Care was taken to choose representative male birds with respect to body weight compared to the group mean body weight. Blood samples (1 ml/bird) were collected into ethylenediaminetetraacetic acid tubes from the wing veins. After centrifugation (3000 rpm, for 10 min at room temperature), plasma was harvested and stored in an Eppendorf tube at −20°C until assayed. Biochemical analysis was according to standard protocols [19] using a commercial analytical kit colesterol end-point enzymatic method, manual form of Randox^®^, according to the manufacturer’s instructions.

### Carcass characteristics parameters

The birds were humanely killed after 42 days of experimental feeding and following the pecking operations with the head and legs separated. Again, care was taken to choose the most representative male birds with respect to body weight compared to the group mean body weight. Weight was recorded for carcass and empty body weight. Weights of the breast, thigh, abdominal fat, liver, pancreas, small intestine, and cecum were recorded too.

### Microbiological evaluation

At the end of the study, i.e., at the 42^th^ day, one bird per group (totally four birds per treatment) was selected for sample collection. Care was taken to choose the most representative male birds with respect to body weight compared to group mean body weight. The cecum content was placed on agar plates for analysis. These samples were also used to determinate bacterial growth and colony counts. Collection tubes were weighted, wrapped in aluminum sheet and autoclaved for 10 min. The culture mediums were prepared and 24 h before collection samples were poured into Petri dishes, Eosin Metilan Blou (EMB,1.01347.0500) was used to culture *E. coli*, according to Espina *et al*. [20].

### Statistical analysis

The study was conducted based on a completely randomized design with four treatments and 10 replicates per treatment. Data were analyzed by InfoStat statistical program using the generalized linear model procedure. The statistical comparison was made by Tukey test at the 95% probability level. The data recorded as ratio or percentages were adjusted within the range between 0% and 30% into their square root. The data recorded as ratio or percentages were transformed into x0.5+0.5. The mathematical model was as follows.

X_ij_=µ+T_i_+ε_ij_

Where, X_ij_=Value observed in each experimental unit, µ=Mean population, T_i_=The effect of each treatment, and ε_ij_=The effect of experimental errors.

## Results and Discussion

### Carcass characteristics parameters

The effect of Ross 308 broilers fed with different treatments on some carcass characteristics from first to 42^th^ day is presented in Table-3. There were five poultry carcass characteristics measured in the experiments, eviscerated carcass, and relative weights of breast, drumsticks (thighs), liver, pancreas, abdominal fat, small intestine, and cecum. However, a simple ANOVA was performed with a level of 95% confidence in the weights obtained from the organs of birds fed with different treatments were compared, in all organs tested a statistically significant difference was obtained except liver. According to the Institute of Nutrition and Food Hygiene, the average liver weight of a bird ranges between 30 and 35 g. The liver is the most voluminous gland present in animal body and plays a fundamental role in the digestion of nutrients, through of the production of bile, liver enzymes, in the metabolism of sugar, proteins, and fats [21]. According to this, we can infer that the treatments have a balance between carbohydrates, proteins and lipids that are within the normal range. For this reason, a food loaded of the lipid could be cause of liver failure and liver fatty (Table-1) [22]. It was observed that the birds with greater weight gain of the eviscerated carcass was the FFC treatment, however an increase considered in the pancreas to unlike the other treatments was observed, due to high glucose concentration in blood causes the pancreas loses its ability to properly handle sugars, gaining weight.

**Table-3:** Carcass yield mean (±SEM) of Ross 40 broilers fed with different agroindustrial waste sampled from postnatal 1^st^-6^th^ weeks*.

Treatment	Eviscerated carcass (g)	Relative weight of breast (g)	Relative weight of drumsticks (thighs) (g)	Relative weight of liver (g)	Relative weight of pancreas (g)	Relative weight of abdominal fat (g)	Relative weight of small intestine (g)	Relative weight of cecum (g)
FC	1463^a^	489.8^a^	391.1^a^	33.4^a^	3.6^a^	9^a^	67.6^a^	14.4^a^
FM	1494^a^	424.4^b^	220^b^	33^a^	5.5^a^	5.6^b^	48^b^	9.6^b^
FFC	1810.1^b^	592.8^c^	212^b^	35.6^a^	3.4^a^	3.6^c^	76.6^c^	9.6^b^
FMW	1718.2^c^	530.7^c^	396.3^a^	34.6^a^	4.4^c^	7.4^d^	21.7^d^	28.6^c^
SEM	146.88	61.27	88.91	2.31	0.82	2.01	21.05	7.78

* Means (±standard error) within each column of dietary treatments with no common superscript differ significantly at p<0.05. FC=Food control, FM=Food+molasses, FFC=Food+ferment of coffee, FMW=Food+milk whey, SEM=Standard error of mean

### Feed intake and weight gain

Avila and Cuca [23], in his article, “sources of energy and protein for feeding birds” concluded that the use of molasses in large quantities causes liquid stool due to the high mineral content; therefore, birds fed the FM treatment did not show a significant effect on weight gain compared to control treatment.

The results obtained by Avila and Cuca [23], coincide with those obtained in this experiment as using food enriched with molasses generates a weight loss due to the production of liquid excrement.

A simple ANOVA at 95% confidence level was performed, considering the data of live weight of control birds about birds of different treatments; the results show a statistically significant difference in the weight of the birds at 22 days of the experiment, demonstrating less weight gain with birds fed the FM treatment. It is concluded that molasses has no significant effect on weight gain of birds, unlike the FFC and FMW treatment that significantly increased the weight of birds (Table-4).

**Table-4:** Performance mean (±SEM) of Ross 40 broilers at starter, finisher and total periods of age fed with different agroindustrial waste sampled from postnatal 1^st^-6^th^ weeks*.

Treatment	Trait		

	Feed intake (g/chick/duration)	Weight gain (g/chick/duration)	Feed conversion ratio
Starter period of age (postnatal 1^st^-21^st^ day)			
FC	920^a^	443^a^	2.07^a^
FM	660^a^	348^a^	1.89^a^
FFC	1001^a^	508^a^	1.97^a^
FMW	1026^a^	511^a^	2^a^
SEM	144.96	66.16	0.064
Finisher period of age (postnatal 22^nd^-35^th^ day)			
FC	2062^a^	1031^a^	1.96^a^
FM	1637^a^	911^a^	1.79^a^
FFC	2767^b^	1536^b^	1.80^a^
FMW	3015^b^	1504^b^	2^a^
SEM	549.04	277.98	0.093
Total period of age (postnatal 1^st^-42^nd^ day)			
FC	3045^a^	1501^a^	2.02^a^
FM	2595^b^	1457^a^	1.78^a^
FFC	3404^a^	1892^b^	1.79^a^
FMW	3821^a^	1949^b^	1.96^a^
SEM	451.73	222.21	0.104

* Means (±standard error) within each column of dietary treatments with no common superscript differ significantly at p<0.05. FC=Food control, FM=Food+molasses, FFC=Food+ferment of coffee, FMW=Food+milk whey, SEM=Standard error of mean

Birds fed with food enriched with molasses showed decreased consumption to unlike other treatments; this result is related to the concentration of blood glucose (269 mg/dl) due to that generated a decrease in carcass weight (Table-5). Because high concentrations of glucose in blood decreases the energy demand, causing a decrease in food consumption, as it has been shown that birds with a concentration of glucose in blood >225 mg/dl have hyperglycemia which stimulates the satiety center [24]. Unlike to the birds fed with whey showed a low level of glucose in blood (194 mg/dL) synonymous with hypoglycemia which stimulates a nerve center for consumption, an aspect that is corroborated by the increased consumption of food at the end of 42 days of treatment with 3821 g (Table-4).

**Table-5:** Biochemical parameters mean (±SEM) of Ross 40 broilers fed with different agroindustrial waste sampled from postnatal 1^st^-6^th^ weeks*.

Treatment	Treat		

	Glucose (mg/dl)	Triglycerides (mg/dl)	Total cholesterol (mg/dl)
FC	220	159.14	247.34
FM	269	231.21	359.04
FFC	249	185.86	288.55
FMW	194	191.66	297.39
SEM	28.4	25.7	39.9

* Means (±standard error) within each column of dietary treatments with no common superscript differ significantly at p<0.05. FC=Food control, FM=Food+molasses, FFC=Food+ferment of coffee, FMW=Food+milk whey, SEM=Standard error of mean

### Biochemical parameters

Saez *et al*. [22] report that a diet with a high content of soluble carbohydrates such as molasses, cause a reduction in pH and generates the relationship of acetate propionate which is essential for the synthesis of glucose in the liver. This explains the difference in the glucose value obtained for the bird in treatment (FM), which was higher compared to bird control.

Konca *et al*. [16] mention that molasses are a source of manganese which helps produce energy and increases good cholesterol indices, including it in the diet; this is consistent with the results obtained in the samples due to that showed higher index of cholesterol in the treatment of birds fed with molasses compared to control treatment. In addition, there is a direct relationship between glucose levels and cholesterol due to that carbohydrate is a source of energy in the liver and it is quickly converted to fat [25,26]. Excessive consumption of fats leads to accumulation of lipids in the hepatocytes, because of this, intake of high-fat diets or energy, are involved in the increase in cholesterol levels [27]. This is consistent with what was observed in the experiment because the food enriched with molasses provides high energy levels that explain the high values obtained for cholesterol.

### Microbiological evaluation

There were three cecum microbial population indexes measured in the experiments, *E. coli*. The statistics of Ross 308 broilers fed with different agroindustrial wastes on counts of cecum microbial population were all significant (Table-6). Due to increase the number of *E. coli*, the counts of *E. coli*, increased significantly (p=0.01 and p=0.02) with the gradual increases of dietary (FM). The results of mean comparisons of *E. coli* in the ileum showed significant differences compared to the control groups. The highest was produced in the FM group and the lowest was related to the FFC treatment of 6.01 log10 cfu/g up to the end of the experimental rearing period.

**Table-6:** Cecum microflora mean (±SEM) of Ross 308 broilers fed with different treatments enriched with agroindustrial wastes from postnatal 1^st^-6^th^ weeks*.

Treatment	Trait

	*E. coli* (log10 cfu/g)
FC	7.01
FM	8.02
FFC	6.10
FMW	6.49
SEM	0.72

* Means (±standard error) within the column of dietary treatments with no common superscript differ significantly at p<0.05. *E. coli=Escherichia* *coli*

## Conclusion

It was shown that the best treatment was the food enriched with the ferment of coffee, due to increased intake (3404 g) and weight gain (1892 g) at the end of 42 days of the experiment, also showing adequate levels of blood glucose (249 mg/dl) and cholesterol (288.55 mg/dl). According to the results of this experiment, it was found that the viability of food enriched with molasses as a diet for poultry is not feasible because the properties of molasses cause hyperglycemia (269 mg/dl) and feces liquid in birds, also does not significantly influence weight gain.

## Authors’ Contributions

EPL and SST designed and supervised the experiment. RBH carried out the experimental work and carried out laboratory analysis of feed samples. YSR and YDCPL did the data analysis and drafted the manuscript. All authors read and approved the final manuscript.
